# Editorial Department

**Published:** 1862-06

**Authors:** 


Fort Edward, Wash. Co., N. Y.

*To the Editor of Buffalo Medical and Surgical Journal:*

While writing my order for your very valuable journal, my eye falls upon a recently published copy of the so called *Electric Medical Journal,* published in Philadelphia, a number of which I have received; and I suppose they are extensively circulated among the regular profession. For what purpose? I ask. This is the question, and one I wish every regular physician in practice to answer in his own mind.

If there is any one thing, more than another, I *detest*, it is quackery in the profession and practice of medicine. I have the most profound abhorrence for all false pretenders, who claim to be making new discoveries in medicine and the healing art. It is quite immaterial what may be the pretention of the quack, we well know that his designs and efforts are to ignore and decry the allopathic system, as it is called, or the regular practice.

What does the regular practice comprehend and embrace? I answer, it comprehends the only rational, safe, and true method of curing disease, while its remedies embrace both the vegetable and mineral kingdoms. It is not limited, but includes all known remedies, and all that may come to the knowledge of man. Nature is its dispensatory; it discards nothing whereupon the visionary and skeptical can speculate.

How grand, noble, and extensive, is this system. The scientific physician is not limited to any particular class of remedies; the great laboratory of nature is before him, and as he is called to administer to suffering humanity, he will select from its store-house such agents as are requisite to combat each successive symptom—this is the great key to success in the practice of physic.

I wish now to ask, what is the electric practice of medicine? It is nothing more, or less, than a dishonest, speculative scheme, to annihilate the regular practice of medicine, making the world believe it ancient and unsafe, and endeavoring to establish another system of practice upon the same basis.

The *Electric Journal* is not only an infamous scandal upon the regular practice, but a libel upon itself. They make use of our most common medicines, and yet curse the regular physician for doing the same. I will refer the profession to no particular number of that journal, as I have seen none but was filled with vile denunciations against the regular system of practice. I denounce it, because it is empirical and dishonest. I am aware that it is circulated among the profession generally; and for the sake of sustaining the character and integrity of our profession, the happiness and well-being of the human family, I trust every member of the profession will do all in his power to resist and put down all such empiricism.

